# Early Breast Cancer Evolution by Autosomal Broad Copy Number Alterations

**DOI:** 10.1155/2022/9332922

**Published:** 2022-02-25

**Authors:** Joseph R. Larsen, Peter Kuhn, James B. Hicks

**Affiliations:** ^1^Convergent Science Institute in Cancer, Michelson Center for Convergent Bioscience, University of Southern California, Los Angeles, CA, USA; ^2^Quantitative and Computational Biology, Department of Biological Science, University of Southern California, Los Angeles, CA, USA

## Abstract

The availability of comprehensive genomic datasets across patient populations enables the application of novel methods for reconstructing tumor evolution within individual patients. To this end, we propose studying autosomal broad copy number alterations (CNAs) as a framework to better understand early tumor evolution. We compared the broad CNAs and somatic mutations of patients with 1 to 10 autosomal broad CNAs against the full set of patients, using data from The Cancer Genome Atlas breast cancer project. We reveal here that the frequency of a chromosome arm obtaining a broad CNA and a genome acquiring somatic mutations changes as autosomal broad CNAs accumulate. Therefore, we propose that the number of autosomal broad CNAs is an important characteristic of breast tumors that needs to be taken into consideration when studying breast tumors. To investigate this idea more in-depth, we next studied the frequency that specific chromosome arms acquire broad CNAs in patients with 1 to 10 broad CNAs. With this process, we identified the broad CNAs that exhibit the fastest rates of accumulation across all patients. This finding suggests a likely order of occurrence of these alterations in patients, which is apparent when we consider a subset of patients with few broad CNAs. Here, we lay the foundation for future studies to build upon our findings and use autosomal broad CNAs as a method to monitor breast tumor progression in vivo to further our understanding of how early tumor evolution unfolds.

## 1. Introduction

In 2020, there were approximately 2.3 million new diagnoses of breast cancer and 685,000 deaths due to the disease worldwide [1]. Current tests and procedures for diagnosing breast cancer are breast exams by a doctor, mammograms, an ultrasound of the breast, or a magnetic resonance image (MRI) of the breast, which are all definitively confirmed by taking a biopsy of the suspected cancerous breast cells [2, 3]. If the biopsy confirms the presence of breast cancer, it is used to identify the stage (i.e. the extent of the cancer) and grade (i.e. proliferation factors and biomarkers like the presence or absence of estrogen, progesterone, and HER2 receptors). The stage, grade, hormone sensitivity, size of tumor, and patient's health are all considered when selecting one or a combination of treatments such as surgery (e.g., removing the cancer, the lymph nodes, or the breast), chemotherapy, radiation therapy, hormone therapy, targeted drug treatments, immunotherapy, or palliative care [2].

Increasing our understanding of breast cancer progression through bioinformatics studies may aid in improving how doctors diagnose, treat, and understand the progression of breast cancer. An example of this is the identification of novel biomarkers. Recent work has used bioinformatics analyses to identify novel biomarkers, such as specific genes and microRNAs that act as targets for breast cancer diagnosis, treatment, and prognosis [4, 5]. These genes and microRNAs may hopefully be able to be used one day in the same way as estrogen, progesterone, and HER2 receptor presence or absence is currently used. Also, researchers have identified microRNAs that are capable of predicting the risk of breast cancer occurrence, efficacy of diet and exercise treatments, and prognosis [6]. Therefore, bioinformatics approaches towards improving diagnosis and treatment for breast cancer patients are promising, but also can deepen our understanding of common occurrences in tumors and possibly improve our capability of predicting what may occur next in breast tumors.

By investigating the relationship between copy number alterations (CNAs) and somatic mutations, we may uncover common patterns that can aid in predicting events for individual patients [7, 8]. CNAs are somatic gains or losses of DNA segments, where narrower ones are defined as focal CNAs and wider ones are defined as broad CNAs [9, 10]. Somatic mutations are changes in a DNA sequence [11]. Both of these genomic events occur after birth and in any cell besides germ line cells, therefore, they are not hereditary [9–11]. Somatic mutations are affected by CNAs and vice versa [12, 13]. Though, CNAs do affect a larger fraction of the genome than somatic mutations and have a recorded history of disease-causing effects [14]. Furthermore, structural variations, such as CNAs, in general, have the potential to affect gene dosage, indirectly affect gene expression, and predispose the genome to experience further structural changes [15], as well as play a key role in tumorigenesis [16]. Finally, previous studies have found that broad CNAs exhibit distinct and frequent patterns in breast cancer genomes [17–22], and probability distributions of CNAs have been used to further understand tumor profiles [10].

For these reasons, we studied the accumulation of broad CNAs, as well as their relationship with somatic mutations, to better understand early tumor evolution. To understand how CNAs progress in breast cancer, under the assumption that CNAs accumulate over time [13], we test whether the frequency distributions of CNAs by chromosome arm change as broad CNAs accumulate [13, 23–30]. Correspondingly, we assume that tumors with a low number of broad CNAs occurred earlier in the tumor progression. Therefore, we tested whether the aggregate profile of broad CNAs and somatic mutations across the tumor genome at the earlier stage of CNA accumulation was distinct from the aggregate profile of tumors with many broad CNAs. A positive result here would indicate that a limited set of broad CNAs and somatic mutations might be considered early events in tumor progression, be associated with cancer initiation, and have distinct properties compared to those CNAs that occur later in the tumor progression. Therefore, we analyzed the frequency profiles of CNAs and somatic mutations in samples from The Cancer Genome Atlas (TCGA), according to the order of event accumulation. Though the overall progression of tumors by CNAs and somatic mutations have been investigated previously [31, 32], our analysis is designed to test whether all events accumulate according to their final frequencies or alternatively, that a subset of events is specific to early stages of the event timeline.

We begin by investigating the qualitative differences between the frequencies of broad CNAs in tumors with ten or less broad CNAs versus the frequencies over all patients. We recognize breast tumors are commonly studied by subtype, but not all the samples in this study in the TCGA were subtyped and therefore we treated them as one group [18–20, 33]. Next, we investigate whether the frequency of a chromosome exhibiting a specific broad CNA is consistent with patients with 10 or less broad CNAs versus all patients. Then, we investigate how the frequencies of a chromosome acquiring a broad CNA and somatic mutations change as broad CNAs accumulate in tumors using the frequencies of broad CNAs for all patients to simulate the corresponding subset of patients with 1 to 10 broad CNAs. Next, we investigate if the trends we have uncovered while considering the number of broad CNAs are consistent with what is known from the literature. Finally, we propose a progression of broad CNAs by chromosome arm for breast tumors with up to 10 broad CNAs in TCGA.

## 2. Materials and Methods

In order to study autosomal broad CNAs, we obtained data from TCGA which we screened and processed prior to being used in our study [34, 35]. This data was used to study trends in broad CNAs found in breast cancer. Due to sex chromosomes having a higher median copy number and variance of alterations [36], we chose to focus on autosomal chromosomes for this study. Also, we studied whether frequencies of broad CNAs change as these alterations accumulate by using frequencies of broad CNAs for all patients, regardless of the number of broad CNAs, to simulate patients with 1 to 10 broad CNAs.

### 2.1. Data Collection

Data for this study was downloaded on March 17, 2020 from TCGA data release 22.0 [35] using the R coding language version 3.5.2 [37] along the *TCGAbiolinks* library version 2.15.3 [38–40]. Specifically, we used the data from the breast tumors in the TCGA project (i.e., TCGA-BRCA). The primary CNA data utilized in this study consisted of 1,089 breast tumor CNA profiles overall, including 180 profiles that have 1 to 10 broad CNAs, and SNV data for 984 breast tumors, including SNV data for 154 breast tumors with 1 to 10 broad CNAs. Of the 1,089 breast tumor CNAs about 1,055 of those patients had at least one type (i.e., normal matched tissue samples and blood-derived normal samples) normal CNA profile, which were also collected. Secondary data included biospecimen and clinical data for screening out duplicate samples and additional supplemental tumor data for subtyping [34]. Centromere locations by base pair were collected using the GRCh38 reference from the Genome Reference Consortium [41]. Finally, we collected supplemental tumor data from additional datasets published by TCGA [34].

An advantage of TCGA for this study is its robust data for hundreds of newly diagnosed, untreated tumors. When cancers are studied in aggregate, there is evidence of characteristic profiles [34, 42–56]. For our purposes here, we used the definition of broad CNAs that states that they are greater than three million base pairs [57, 58]. We assume that the breast tumors analyzed for TCGA are undergoing the same stochastic evolutionary process, and that they represent cross-sectional data taken from a single biopsy, prior to treatment, of independent tumors [23, 35, 59–62]. Using this breast cancer data here, we investigated whether certain events may occur more frequently in the emergence of tumors.

### 2.2. Data Processing

#### 2.2.1. Selecting Somatic Variant Caller

The somatic variant callers available on TCGA as of March 17, 2020, include SomaticSniper, Varscan2, MuSE, and Mutect2. Based on studies regarding the performance of these four somatic variant callers, we elected to use the output of the Mutect2 for this study [63–65].

#### 2.2.2. Removing Duplicate Samples

Cancer CNA data (1,089 profiles) along with their respective matched normal CNA profiles (1,055 profiles) were downloaded for all patients in TCGA-BRCA [34]. For cancer CNA samples, we removed duplicates in the following order of priority: if the sample was Formalin-Fixed Paraffin-Embedded (FFPE), if a duplicate had a later date of creation, if a duplicate had a later date of shipment, and if it was used in any other additional project in TCGA. The removed cancer CNA samples, by unique TCGA barcodes, were TCGA-A7-A0DC-01B-04D-A22Q-01, TCGA-A7-A13G-01B-04D-A22Q-01, TCGA-A7-A26E-01A-11D-A275-01, TCGA-A7-A26E-01B-06D-A275-01, TCGA-A7-A26F-01B-04D-A22Q-01, TCGA-A7-A26J-01A-11D-A275-01, TCGA-A7-A26J-01B-02D-A275-01, TCGA-AC-A2QH-01B-04D-A22Q-01, TCGA-AC-A3OD-01A-11D-A21P-01, and TCGA-B6-A1KC-01A-11D-A13J-01.

The same criteria were used when processing normal CNA samples, with an additional three criteria. Duplicates were saved if they were of a different type (i.e., normal matched tissue samples and blood-derived normal samples). So, if a patient had a single normal matched tissue sample and blood-derived normal sample, neither were removed and both were labeled by sample type. Second, if a normal sample appeared contaminated, we removed the sample even if it was the only normal sample a patient had. Finally, samples were removed if there was not a corresponding cancer CNA sample. The removed normal CNA samples unique TCGA barcodes were TCGA-A7-A26E-10A-01D-A275-01, TCGA-A7-A26J-10A-01D-A275-01, TCGA-E2-A1LS-11A-32D-A160-01, TCGA-A7-A0DC-11A-41D-A092-01, TCGA-BH-A0BW-11A-12D-A111-01, TCGA-BH-A0H7-11A-13D-A092-01, TCGA-E2-A158-11A-22D-A12A-01, TCGA-E2-A15L-11A-31D-A12A-01, TCGA-BH-A0DD-11A-23D-A12N-01, TCGA-E2-A1LI-11A-23D-A160-01, TCGA-BH-A18M-11A-33D-A12A-01, TCGA-AC-A2QJ-11A-12D-A19X-01, TCGA-AC-A2BM-11A-13D-A21P-01, TCGA-A7-A0CH-11A-32D-A092-01, TCGA-BH-A1EX-11A-21D-A13N-01, and TCGA-BH-A1EW-11B-33D-A134-01.

#### 2.2.3. Data Analyses prior to Download

After screening the samples, we needed to identify somatic CNAs. The open-access CNA data available by TCGA was obtained after being derived from Affymetrix 6.0 single nucleotide polymorphism (SNP) arrays and noise-reduction via Circular Binary Segmentation (CBS). After cancer DNA was analyzed for gains or losses compared to normal DNA for each probe, corresponding to a SNP with a specific location in the genome, the CBS algorithm creates segments of probes that are significantly different [66, 67]. We identified these segments to be in regions of genome gain, genome loss, or the sample's baseline (i.e., the region of the genome with no gains or losses). In order to identify a sample's somatic CNAs, we first identified the samples' baselines in order to determine the threshold values that indicate a gain and a loss. We identified each patient's baseline separately, because baselines were found to vary between patients. Additionally, technical noise had resulted from data analyses prior to download. Similarly, each sample's threshold for gains and losses is different, because each sample has different levels of normal cells and heterogeneity present.

#### 2.2.4. Approximating CNA Baselines

We first approximated the baseline for CNA samples. We began with the assumption that a majority of the genome is not gained nor lost [9]. We then considered all combinations of segments that represent a majority of the genome by base pair (e.g., up to 51%, of base pairs) and found the mean and standard deviation for each group of segments. The group of segments with the lowest standard deviation was believed to comprise the baseline, and the average of these segments was used to approximate the baseline for a CNA sample.

#### 2.2.5. Deriving CNA Gain and Loss Thresholds

Once we had an approximate baseline, we needed to decide at what value above and below a baseline a segment is considered a gain or loss, respectively. There were difficulties due to the fact that the tumor samples have varying degrees of noncancer cells, which is inconsistent between patients and causes diluted cancer CNA signals. We began by finding a gain threshold and loss threshold that would exclude all segment heights in the baseline, when considering the variation of segment heights in the group. We created these thresholds by first collecting the segments of all normal cells that were greater than 3,000,000 base pairs, because CNAs are rare in normal cells and broad alterations are even rarer. Here, we used the definition of broad CNAs being over 3,000,000 base pairs long [9, 57, 58]. As a result, removing these segments leaves only the few alterations that vary the most from segments in the baseline. So, we next removed any outlier segment heights from this collection of segments. This collection is what we assumed segments without gains or losses are most similar to. For each sample, we approximated the baseline of this entire collection of segments from all normal samples. We added the maximum segment height of the remaining collected segments and the corresponding standard deviation to the approximate baseline to create a conservative gain threshold. Similarly, we subtracted the minimum segment height of the remaining collected segments and the standard deviation between these segment heights from the approximate baseline to create a conservative loss threshold. These thresholds represent how far a segment height has to be from the approximate baseline to be considered a gain or loss and is applied to all samples when screening for alterations.

To account for technical noise, we determined the approximate baseline of each sample and the standard deviation of the segment means in that group, and then we added the standard deviation to both the gain threshold and loss threshold. Then, to combat noise due to heterogeneity, any alteration that was less than one-eighth of the most extreme segment mean, either the maximum gain or minimum lost, was assumed to not be part of the major clonal population. As a result, they were removed and not considered for our analyses. Consecutive alterations (i.e., alterations that occur without returning to the baseline between alterations) were recognized if the segment was approximately one-fourth of the extreme segment mean more or less than the neighboring segments. These methods were applied to chromosomes of each patient CNA sample to recognize CNAs and acquire a sum of autosomal broad CNAs.

#### 2.2.6. CNA Data Visualization

All plots of data that have undergone preprocessing presented in this article were created using the base graph functions found in the R language version 3.5.2 [37]. The R library scales version 1.1.0 was used for introducing various color options to present chromosome arm alterations together on one plot [68]. Trends displaying the frequencies of patients with a specific alteration by chromosome, on the *y*-axis, over number of autosomal broad CNAs, on the *x*-axis, were depicted using local regression with an automatic smoothing parameter selection (i.e., Akaike information criterion) which was accomplished by the *loess.as* function found in the *fANCOVA* library version 0.5-1 for R [69].

### 2.3. Simulating Patients by Event Counts of Subsets of Patients from Event Frequencies of All Patients

To test whether we could approximate the frequencies of broad CNAs for patients with 1 to 10 broad CNAs using the frequencies of CNAs from all, we simulated patients with 1 to 10 broad CNAs using the frequencies of broad CNAs from all patients. We began by sampling all patients by calculating their cumulative frequency by broad CNAs over all chromosome arms. We generated a random value between 0 and 1 using the uniform distribution to represent each autosomal broad CNAs in each patient in the subset. From the value generated, we assigned the broad CNA based on the value in relation to the distribution of cumulative frequencies. Once we have the same number of simulated patients as the number of patients in the subset, that iteration is complete. This process is repeated for 1,000 iterations. Though we recognize this manner of generating simulated patients may produce a patient with both a gain and loss on the same chromosome arm, this scenario is unlikely, and if it does occur, it would only introduce minimal amounts of error. Finally, we chose to compare the frequencies for all patients to those of the subset of patients over various intervals of broad CNAs and the simulated patients over the same intervals of broad CNAs, using the Kendall Correlation. Kendall was preferred over Pearson due to the presence of outliers and over Spearman due to the robustness and efficiency of Kendall in comparison [70]. This process is repeated for the somatic mutations of patients over various intervals of broad CNAs.

## 3. Results

### 3.1. Autosomal Broad CNAs in Breast Cancer Patients

**Figure 1:**
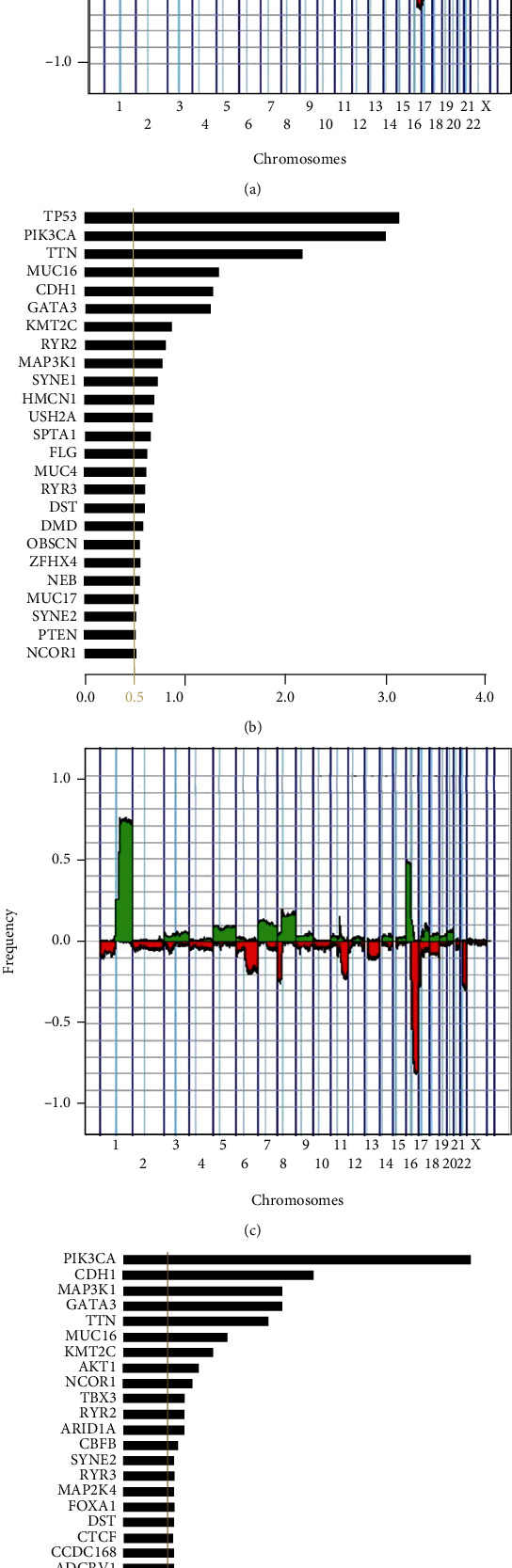
Frequency of all patients by genomic events. (a) Frequency of all patients (1,089 patients) gains (greater than 0 and green) and losses (less than 0 and red) for each SNP location on the Affymetrix SNP6.0 Microarray. (b) Frequency of all patients (984 patients) somatic mutations for any somatic mutation present in over 5% of all patients. (c) Frequency of the gains (greater than 0 and green) and losses (less than 0 and red) in patients, with 1 to 10 autosomal broad CNAs (180 patients), for each SNP location on the Affymetrix SNP6.0 Microarray. (d) Frequency of somatic mutations for any somatic mutation present in over 5% of patients in the subset in patients, with 1 to 10 autosomal broad CNAs (154 patients).

We began our study by visualizing the most common CNAs, both broad and focal, and somatic mutations among 1,089 patients in the breast cancer project of the TCGA and TCGA-BRCA (Figures 1(a) and 1(b)). The most common CNA is the chromosome 1 arm q broad gain (1qG), which nearly encompasses the entire arm and occurs in approximately 80% of all patients, whereas the most common somatic mutation, the mutation to the TP53 gene, occurs in less than 40% of the 984 patients with somatic variant caller data of the 1,089 patients curated here. Therefore, we suggest that the most frequent events that occur between all patients are broad CNAs as opposed to any other CNAs and somatic mutations.

We then turned our focus to patients with 1 to 10 autosomal broad CNAs to investigate whether a difference exists among frequencies of CNAs and somatic mutations when compared to observing all patients regardless of the number of broad CNAs. To this end, we extracted the group of patients with 1 to 10 autosomal broad CNAs from all patients in the TCGA-BRCA and identified the frequency of patients with copy number alterations (i.e., 180 patients) and somatic mutations (i.e., 154 patients) across their genome (Figures 1(c) and 1(d)). We found that the chromosome 16 arm q broad loss (16qL) is the most common CNA and occurs in approximately 80% of patients in the subset. The most frequent somatic mutation among patients in the subset with somatic variant caller data is PIK3CA at less than 40%, which is also 10% more common than any other somatic mutation among patients with 1 to 10 broad CNAs in TCGA-BRCA.

We then examined the specific similarities and differences between the frequencies of patients in the subset exhibiting 1 to 10 broad CNAs and frequencies of all patients. One similarity is the high frequency of the chromosome 16 arm p broad gain (16pG), 1qG, and 16qL in the two plots (Figures 1(a) and 1(c)). In addition, the chromosome 8 arm p broad loss (8pL), chromosome 8 arm q broad gain (8qG), chromosome 11 arm q broad loss (11qL), chromosome 17 arm p broad loss (17pL), and chromosome 22 arm q broad loss (22qL) exhibit higher frequencies in both the subset of 1 to 10 broad CNAs and all patients as opposed to other CNAs. Also, we see that there are alterations with a frequency that is almost as low as 0 in the subset of patients with 1 to 10 broad CNAs, but these same alterations have frequencies noticeably larger in the group of all patients. Considering the assumption that alterations accumulate over the lifetime of the tumor [13], these alterations are thought to be later broad alterations of breast cancer, which is why they do not appear in the subset of patients with 1 to 10 broad CNAs. Most notably, PIK3CA has the highest frequency among patients in the subset with 1 to 10 broad CNAs but is second only to TP53 among frequencies for all patients. This suggests that patients who acquire the mutation to the PIK3CA gene are more likely to acquire it early on in the development of their tumor, but the mutation to the TP53 mutation tends to occur later and possibly at a higher rate.

### 3.2. The Frequency of Broad CNAs and Mutations Change as Autosomal Broad CNAs Accumulate

**Figure 2:**
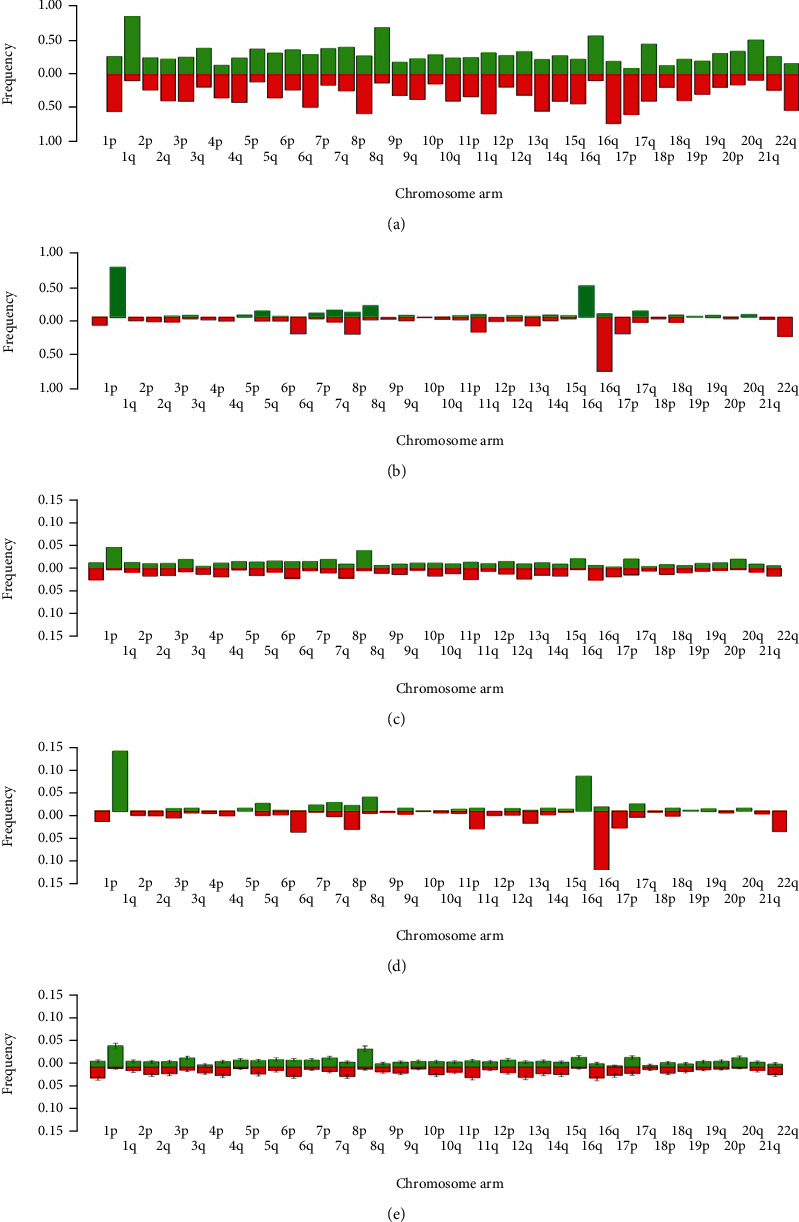
Frequencies by chromosome arm. (a) Frequency of all patients that exhibit broad CNAs by chromosome arm. (b) Frequency of patients with 1 to 10 autosomal broad CNAs that exhibit broad CNAs by chromosome arm. (c) Frequency of broad CNAs of all patients by chromosome arm. (d) Frequency of broad CNAs of patients with 1 to 10 autosomal broad CNAs by chromosome arm. (e) Average frequency of broad CNAs, with error bars representing 1 standard deviation, from 1,000 iterations of randomly sampling all patients to recreate the number of broad CNAs in patients with 1 to 10 autosomal broad CNAs by chromosome arm.

Because our results indicated that the most frequent broad CNA was 16qL in the subset of patients with 1 to 10 autosomal broad CNAs and 1qG in all patients, we next chose to investigate if the frequency a chromosome arm acquires a broad alteration changes as the genome acquires more broad alterations overall. Keeping our focus on autosomal broad CNAs, we re-binned the data by chromosome arm as opposed to SNP location for all patients (Figure 2(a)) and for the subset of patients with 1 to 10 broad CNAs (Figure 2(b)). We found the frequency of patients with a broad alteration on each chromosome arm for both groups is consistent with the patterns prior to rebinning.

We then investigated the frequency of autosomal broad CNAs by chromosome arm, versus the frequency of patients by chromosome arm above (Figures 2(a) and 2(b)), for all patients (Figure 2(c)) and the patients in the subset of 1 to 10 broad CNAs (Figure 2(d)). The frequency of autosomal broad CNAs in all patients appears to have a nearly uniform distribution between arms, while the frequency of autosomal broad CNAs in the subset of patients with 1 to 10 broad CNAs displays certain arms experiencing broad alterations more than others (e.g., 1qG, 16pG, and 16qL). So, we next tested whether the distribution of the frequency of autosomal broad CNAs for all patients (Figure 2(c)) is approximately the population proportions of the distribution of frequency of autosomal broad CNAs for the subset of patients (Figure 2(d)). If the frequency of autosomal broad CNAs for all patients is approximately the population proportion, then, these autosomal broad CNA frequencies should be able to simulate all 180 patients in the subset and their respective number of autosomal broad CNAs (totaling 1,150 autosomal broad CNAs) and to acquire a distribution that is approximately the same as the patients with 1 to 10 broad CNAs (Figure 2(d)).

We begin by hypothesizing the distribution of frequencies of broad CNAs for all patients (Figure 2(c)) is the same no matter the total number of broad CNAs over a subset. Then, we randomly generated broad CNAs, using the frequencies of autosomal broad CNAs in all patients by chromosome arm, to simulate the number of broad CNAs of each patient with 1 to 10 broad CNAs. We repeat this process 1,000 times and then average the frequencies of autosomal broad CNAs over all 1,000 iterations (Figure 2(e)). This distribution more closely matches the distribution of frequencies of broad CNAs for all patients as opposed to the frequency of broad CNAs for the subset of patients with 1 to 10 broad CNAs. We found that the Kendall correlation between the mean frequencies of the simulated distribution of patients with 1 to 10 broad CNAs and all patients is 0.994 versus 0.544 when correlated with patients with 1 to 10 broad CNAs in the subset. Also, the correlation between all patients and the subset of patients with 1 to 10 broad CNAs is 0.546. The correlation values are very similar for the comparison between the subset of patients with 1 to 10 broad CNAs to all patients (0.546) and the subset of patients to the simulated patients (0.544). Because the frequencies of autosomal broad CNAs for all patients were unable to accurately simulate patients with 1 to 10 broad CNAs with a similar number of alterations for the same number of patients, we reject that the distribution of frequencies of autosomal broad CNAs for all patients is approximately the population proportion. Therefore, we propose the frequency of broad alterations change as the number of broad alterations accumulates.

We then repeated the same test for frequencies of somatic mutations. Similarly, we hypothesized that the distribution of frequencies of somatic mutations for all patients is the same no matter the number of autosomal broad CNAs over a subset. We repeated the same simulation as above, now using the frequencies of somatic mutations to randomly generate somatic mutations for each patient, and their respective number of somatic mutations, in the subset of patients with 1 to 10 broad CNAs. We repeated this process 1,000 times and averaged the frequency for each somatic mutation. The Kendall correlation between the mean frequencies of somatic mutations for the simulated distribution of patients with 1 to 10 broad CNAs and all patients is 0.965 versus 0.463 when correlated with the frequencies of somatic mutations for patients with 1 to 10 broad CNAs in the subset. The correlation is 0.480 for all patients and the subset of patients, which is much more similar to the correlation of simulated patients and the subset of patients. So, we once again reject the hypothesis that the distribution of frequencies of somatic mutations for all patients is approximately the population proportion. Therefore, we propose the frequency of somatic mutations change as the number of broad alterations accumulates.

### 3.3. Broad CNA and Mutation Frequencies Vary with Few Autosomal Broad CNAs

**Figure 3:**
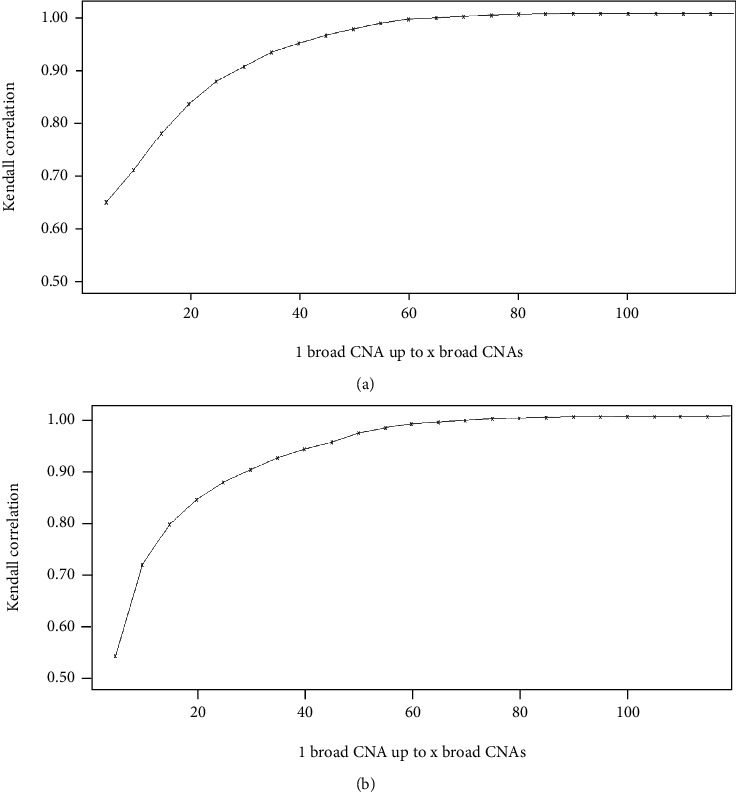
Kendall correlations between subset of patients and simulated patients. (a) The correlation between the broad CNAs by chromosome arm of the subset of patients with 1 to *x* autosomal broad CNAs, where *x* is the maximum number of autosomal broad CNAs in the interval and the corresponding simulated subset of patients. (b) The correlation between the somatic mutations of a subset of patients with 1 to *x* autosomal broad CNAs, where *x* is the maximum number of autosomal broad CNAs in the interval, and the corresponding simulated subset of patients.

Next, we investigated the change of frequencies of autosomal broad CNAs and somatic mutations in a subset of patients with an increasing number of events (i.e., 1 to 5, 1 to 10, and 1 to 15), and we determine whether these groups correlate to the results from all patients or if they gradually converge to the pattern in all patients as more autosomal broad CNAs are included. To this end, we ran the same simulation as above for subsets of patients with 1 to 5, 1 to 10, 1 to 15, and so on up to 1 to 120 broad CNAs. We then calculated the Kendall correlations for each simulated subset and the actual subset of patients with the respective interval of autosomal broad CNAs for both broad CNAs and somatic mutations (Figures 3(a) and 3(b)). In the plot of Kendall correlations over increasing subset size (Figure 3(a)), we show that the correlations converge to one as the interval increases. Therefore, there is not a random or discrete growth of correlation as the intervals of broad CNAs of patients increase, but instead, more of a continuous growth as the interval expands. Biologically, this is significant, because we can see that if we analyze tumors without considering the number of autosomal broad CNAs, we may make conclusions about aspects of a tumor type, such as what are the frequencies of alterations and mutations, which are not consistent for tumors with fewer number of autosomal broad CNAs. The gradual increase of correlation as the subset increases allows us to recognize at what upper limit of autosomal broad CNAs the frequencies begin to appear similar to all patients regardless of the number of broad CNAs versus intervals with up to 5 or 10 autosomal broad CNAs.

The highest rate of change is among subsets with a lower upper bound of autosomal broad CNAs, and gradually, the correlation becomes more similar to all patients when the maximum number of autosomal broad CNAs is over 30. Once the subset has 1 to 30 broad CNAs and higher, the correlation is over 0.900 and becomes approximately 1.000 when the subset has 1 to 80 broad CNAs and higher. After the upper limit of autosomal broad CNAs in our interval reaches 30 broad CNAs, the frequencies of chromosome arms with broad CNAs are more correlated with all patients regardless of the number of broad CNAs versus the intervals up to 5 or 10 broad CNAs. Overall, the curve approaches a correlation of 1.000 logarithmically as the interval grows to include all patients.

Next, we plotted the Kendall correlations over increasing subset size of the frequency of somatic mutations (Figure 3(b)), and we found that these correlations also converge to one as the interval increases. Similar to that of broad CNAs, we see a continuous growth, as opposed to random or discrete growth, between intervals of subsets of autosomal broad CNAs. Again, we were curious at what upper limit of autosomal broad CNAs do the frequencies of somatic mutations appear more like all patients regardless of the number of broad. We found that once the subset has 1 to 30 and more autosomal broad CNAs, the correlation exceeds 0.900. Once again, when the upper limit of broad CNAs in our interval reaches 30 broad CNAs, it appears that the frequencies of chromosome arms with broad CNAs are more correlated with all patients than the groups of intervals up to 5 or 10 broad CNAs. The key difference is that the rate of change between the correlations as the subsets grow seems to be faster for the frequencies of somatic mutations than frequencies of broad CNAs, because the correlation is approximately 1.000 when the subset has 1 to 70 broad CNAs and higher. This difference may be attributed to the greater variability of the number of autosomal broad CNAs or the larger number of somatic mutations versus autosomal broad CNAs. Once again, the curve approaches a correlation of 1.000 logarithmically as the interval grows to include all patients.

### 3.4. Validating Early Alterations When Considering Number of Autosomal Broad Alterations

Based on our findings above, we chose to move forward by not considering autosomal broad CNAs from all patients all together but instead from patients with few autosomal broad CNAs. These patients with fewer broad CNAs appear to have the most different distribution of broad CNAs and somatic mutations (Figure 3) from patients who have more mature tumors with more broad CNAs. Therefore, there are distinct characteristics of a tumor based on the number of broad CNAs, further illustrating the need for autosomal broad CNAs to be considered when studying tumors. To this end, we continued by focusing on patients with only two autosomal broad CNAs, to validate if what we find in patients of the TCGA-BRCA project by the number of autosomal broad CNAs aligns with known early breast tumor events presented in the literature.

**Figure 4:**
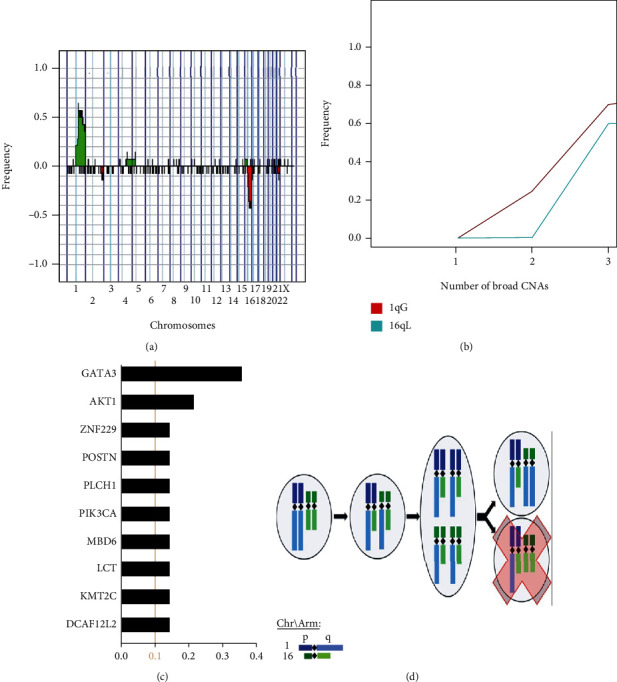
Initial two events in most breast cancer patients appear to be 1qG and 16qL. (a) Frequency of gains (greater than 0 and green) and losses (less than 0 and red) of patients, with 1 to 2 autosomal broad CNAs (14 patients), for each SNP location on the Affymetrix SNP6.0 Microarray. (b) Frequency of patients with 1qG (cyan) and 16qL (red) by number of autosomal broad CNAs. (c) Frequency of somatic mutations for any somatic mutation present in over 10% of patients in the subset of patients, with 1 to 2 autosomal broad CNAs (14 patients). (d) The fewest structural variation events required for a full arm centromere recombination-based alteration to create a 1qG and 16qL in a normal breast cell.

Previously, researchers have been able to capture and record early CNAs in breast cancer cells by observing the variation mid-alteration [71–73]. When studying these cells, they consistently recorded two initial alterations, 1qG and 16qL. Therefore, we decided to examine if patients in the TCGA-BRCA exhibit these broad CNAs at high frequencies when there are 2 or less autosomal broad CNAs present. First, we studied the frequency of patients with 2 or less autosomal broad CNAs with an alteration for each SNP location on the Affymetrix SNP6.0 Microarray (Figure 4(a)). There are only 14 patients with 2 or less autosomal broad CNAs present in the TCGA-BRCA, which aligns with our assumption that alterations accumulate as the tumor matures, because an individual would have to recognize they have a tumor and have a biopsy of their tumor analyzed in its earlier stages for us to have such data. That being said, we use this dataset of 14 patients to compare to what was seen previously in the literature. Over half of the patients have 1qG and over 40% have 16qL which are five and four times more than any other alterations present, respectively. We see that 25% of patients with 1 broad CNA (i.e., 1 patient out of 4 with 1 autosomal broad CNA) acquired 1qG as their only alteration and then 70% of patients with 2 broad CNAs (i.e., 7 patients out of 10 with 2 autosomal broad CNAs) have a 1qG (Figure 4(b)). Alternatively, the frequency of 16qL is 0% in patients with 1 broad CNA and is 60% at 2 broad CNAs (i.e., 6 patients out of 10 with 2 autosomal broad CNAs). This result indicates that only patients with 2 broad CNAs exhibited 16qL in this group, and it occurs at a high frequency (Figure 4(b)). Our results here align with previous findings that 1qG and 16qL most commonly occur together and early [71–73].

Only ten somatic mutations occurred in at least 2 of the 14 (i.e., over 10%) patients with 1 or 2 broad CNAs (Figure 4(c)). The two most common mutations exhibited among these alterations were AKT1, with 3 patients, and GATA3, with 5 patients. Although GATA3 has been recognized as a prominent mutation in breast cancer [34], it still occurs at a lower frequency than both 1qG and 16qL in this subset.

We hypothesize here how a normal cell may acquire 1qG and 16qL, which may be the first two alterations leading to breast cancer. Following an Occam's Razor approach, we considered what would be the lowest number of genomic imbalance events a normal cell would exhibit to acquire 1qG and 16qL. The fewest number of events possible is two. These events would occur during mitosis in which first a translocation of the q arms of an allele of chromosome 1 and an allele of chromosome 16 occur followed by a separation error during division (Figure 4(d)). The separation would produce one daughter cell with the correct number of p arms for both chromosome 1 and chromosome 16 but an extra q arm of chromosome 16 and a missing q arm of chromosome 1. However, this daughter cell is not commonly observed. We believe this may be due to this daughter cell dying or not proliferating to a level we can recognize it. The other daughter cell becomes the CNA profile with the proposed initial broad alterations, 1qG and 16qL, found at a high frequency here and previously in the literature [71–73].

### 3.5. Ordering Autosomal Broad CNAs of Patients with up to 10 Autosomal Broad CNAs

Here, we expand upon our findings above and propose other early alterations that may occur in breast tumors and in what order in terms of the number of autosomal broad CNAs. We group patients by increasing number of autosomal broad CNAs, up to 10 broad CNAs, and investigate the trends in frequency of broad CNAs by chromosome arm that are most frequently found in patients with less than 10 broad CNAs. By recognizing these trends, we propose a possible order for what chromosome arms acquire broad CNAs.

**Figure 5:**
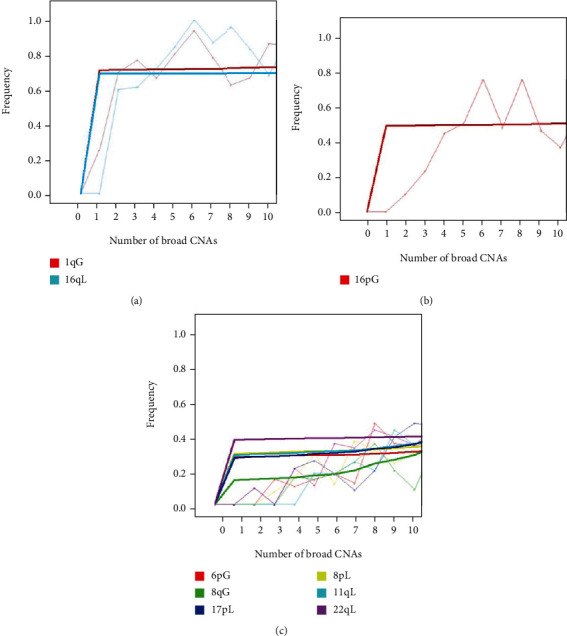
Frequency of patients by number of autosomal broad CNAs from 1 to 10 by chromosome arm and type of alteration. Lighter colored lines represent raw data while bolder colored lines represent trends of the data, fitted by local regression, for each plot. (a) Frequency of patients by number of autosomal broad CNAs for chromosome arm alterations greater than approximately 80% of all patients with 1 to 10 autosomal broad CNAs. (b) Frequency of patients by number of autosomal broad CNAs for chromosome arm alterations greater than approximately 40% but less than approximately 50% of all patients with 1 to 10 autosomal broad CNAs. (c) Frequency of patients by number of autosomal broad CNAs for chromosome arm alterations greater than approximately 20% but less than approximately 30% of all patients with 1 to 10 autosomal broad CNAs.

We begin our analysis of frequency of patients with broad CNAs by chromosome arm by only considering chromosome arms that have alterations in more than 80% of patients with 1 to 10 autosomal broad CNAs. The only two chromosome arms with broad CNAs with such a high frequency are 1qG, with 134 patients out of 180 patients with 1 to 10 broad CNAs, and 16qL, with 143 patients out of 180 patients with 1 to 10 broad CNAs (Figure 1(c)). The trends of frequencies (the bold lines) for the raw frequencies (the faded lines) of broad CNAs by chromosome arm over the number of broad CNAs of the patient (up to 10) rapidly converge around 2 broad CNAs (Figure 5(a)). Hence, these frequencies tend to occur before other broad CNAs in this dataset, remaining consistent with our findings above.

Next, we turn our focus to the frequent broad CNA among patients, after 1qG and 16qL, by chromosome arm for chromosome arms that have alterations in approximately 50% of patients with 1 to 10 autosomal broad CNAs (Figure 5(b)). The only chromosome arm, not previously discussed, to experience a broad alteration among this frequency of patients is 16pG, which 85 patients out of 180 patients with 1 to 10 broad CNAs exhibit (Figure 1(c)). Although the trend (the bold lines fitted by local regression) seems to converge early at approximately 50% of broad CNAs by chromosome arm, we can see that the raw data (the faded lines) appears to indicate a rise at a slower rate than 1qG and 16qL (Figure 4(a)). This suggests patients with 1 to 10 broad CNAs acquire 16pG at a slower rate than 1qG and 16qL, and therefore, 16pG occurs after 1qG and 16qL.

Lastly, we bring our attention to the most frequent broad CNAs among patients, after 1qG, 16qL, and 16pG, by chromosome arm for chromosome arms that have alterations that are approximately within 20% to 30% of patients with 1 to 10 broad CNAs (Figure 5(c)). This group has six chromosome arms with broad CNAs for the 180 patients in this subset: chromosome 6 arm q broad loss (6qL) in 43 patients, 8qG in 32 patients, 8pL in 44 patients, 11qL in 38 patients, 17pL in 43 patients, and 22qL in 51 patients (Figure 5(c)). The raw data (the faded lines) depict all five gradually increase their frequency of patients over the number of broad CNAs. Once we fit this raw data to their respective trend lines (the bold lines fitted by local regression), we see the trends suggest an order of occurrence for these broad CNAs, which is first 22qL, then, either 6qL, 8pL, 11qL, or 17pL in what currently appears to be an undiscernible order, and finally 8qG.

## 4. Discussion

**Figure 6:**
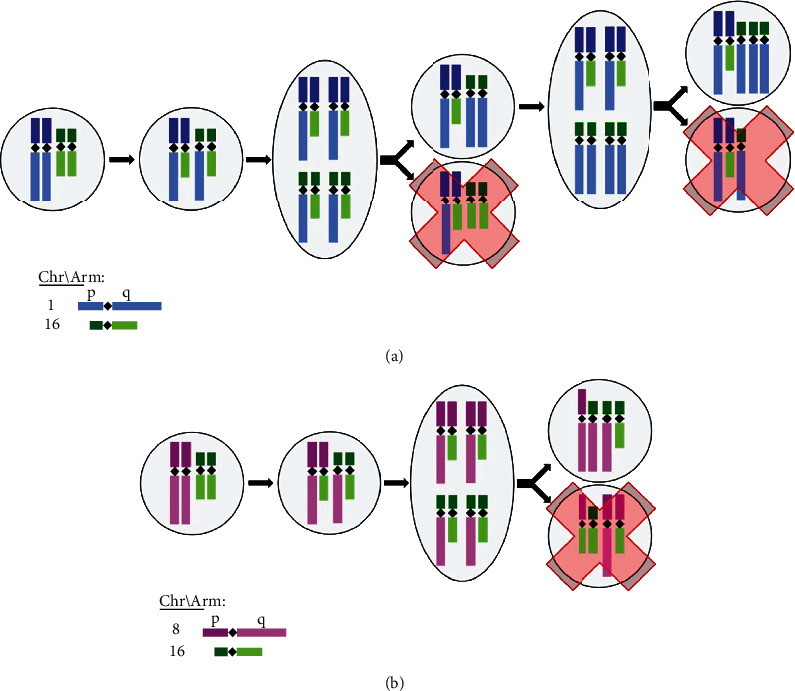
Mechanism leading to patterns of broad copy number alterations in breast cancer. (a) The fewest structural variation events required for a full arm centromere recombination-based alteration to create 1qG, 16pG, and 16qL. (b) The fewest structural variation events required for a full arm centromere recombination-based alteration to create 8qG, 8pL, 16pG, and 16qL.

We have found here that the distributions of broad CNAs change as autosomal broad CNAs accumulate and demonstrated that the frequency profiles of broad CNAs and somatic mutations in tumors early in their progression are distinct compared to the aggregate profiles of more genomically complex tumors. We interpret this to mean that broad CNAs and somatic mutations do not occur randomly during tumor progression, but instead present in generally predictable patterns. Our results further demonstrate that the most frequent early alterations in the breast tumors are 1qG and 16qL followed by 16pG. We expanded the proposed mechanism of how a normal cell initially acquires 1qG and 16qL by adding the next alteration, 16pG (Figure 6(a)). Only one structural variation event is required, an additional separation error, for 16pG to occur after 1qG and 16qL. After 16pG, we found that the most likely alterations are 6qL, 8pL, 8qG, 11qL, 17pL, and 22qL, with a possible order, from raw data, of 22qL followed by 6qL, 8pL, 11qL, and 17pL in no discernable order and then finally 8qG. The trends for 6qL, 8pL, 8qG, 11qL, and 17pL appear to differentiate the most around 10 broad CNAs, possibly indicating something inherent to breast tumors changing the order these CNAs occur.

We found that the relationship between the number of autosomal broad CNAs and molecular subtypes follow commonly observed patterns of CNAs in breast cancer [18–20, 33]. Of the 74 patients with 1 to 10 autosomal broad CNAs who have known molecular subtypes (out of 180 patients with 1 to 10 autosomal broad CNAs), 60 of them are Luminal A (i.e., 81%), 11 of them are Luminal B (i.e., 15%), 0 of them are HER2-enriched (i.e., 0%), 2 of them are Normal-like (i.e., 3%), and 1 is Basal-like (i.e., 1%). This group is dominated by Luminal A tumors, which may be due to Luminal A tumors having low histological grade, low gene proliferation, and a good prognostic among these subtypes [33]. Due to these characteristics, Luminal A tumors may be more easily detected at an earlier state with 10 or fewer broad CNAs. Also, a common pattern of CNAs in Luminal A tumors is 1qG and 16qL, which validates our findings here [22]. In contrast, of the 447 patients with greater than 10 broad CNAs who have known molecular subtypes (out of 909 patients with greater than 10 autosomal broad CNAs), 171 of them are Luminal A (i.e., 38%), 116 of them are Luminal B (i.e., 26%), 57 of them are HER2-enriched (i.e., 13%), 6 of them are Normal-like (i.e., 1%), and 97 of them are Basal-like (i.e., 22%). All subtypes besides Luminal A and Normal-like see an increased frequency, with Basal-like tumors having the greatest increase. This aligns with our understanding of Basal-like tumors, because they have high histological grade, high gene proliferation, and a poor prognosis among these subtypes [33]. Accordingly, Basal-like tumors are more difficult to detect with 10 or fewer broad CNAs. The change in distribution of molecular subtypes could explain why the trend lines for 6qL, 8pL, 8qG, 11qL, and 17pL change around 10 broad CNAs. Future work will seek to separate patients by classifications of breast cancer, such as molecular subtypes, to investigate if likelihoods of chromosome arms obtaining broad CNAs change by classifications and possibly add new perspective to these classifications [74].

Future work includes refining and improving the algorithm we developed and used to identify autosomal broad CNAs as newer data, tools, and methods are released. Furthermore, we plan to study all chromosome arms' likelihoods of broad CNAs, and which chromosome arms' frequencies trend together. One way to do this would be to adapt longitudinal data clustering techniques, in which we treat the number of autosomal broad CNAs as temporal data and use a parameterization that clusters over frequency and number of autosomal broad CNAs [75]. Lastly, we will model the progression of disease [7, 76, 77, 78] using cross-sectional data [23, 59–62] to find likelihoods for the order of onset of broad CNAs in breast cancer.

One limitation of our study is that our findings need to be validated by independent datasets to confirm our conclusions. A possible dataset is the Molecular Taxonomy of Breast Cancer International Consortium [20], which requires application for access, because it is controlled data. Also, we used the output of somatic variant caller Mutect2, because it outperformed the other variant callers available [63–65], but a combination of other somatic variant callers available from TCGA may produce more accurate depictions of somatic mutations [79, 80]. If so, our results should be tested against these findings. Our results may also be improved by using newer data that was collected and processed by newer technology for identifying autosomal broad CNAs. The CNAs detected in the tumors used in this study came from microarrays, specifically the Affymetrix SNP 6.0 [66], but using next-generation sequencing (NGS) to detect CNAs is preferable because of the advantages of being able to better recognize novel CNAs as well as increased accuracy in estimating copy numbers [81, 82]. Therefore, our conclusions should be tested specifically against data collected from NGS.

We recognized a majority of patients appear to first obtain 1qG and 16qL, which is consistent with previous findings even though there have been other common, but less frequent, initial alteration patterns in breast cancer. Specifically, the second most common initial alteration pattern in breast cancer patients is 8pL, 8qG, 16pG, and 16qL [17]. We hypothesize that the simplest mechanism of the full arm centromere recombination-based alteration for 8pL, 8qG, 16pG, and 16qL would be similar to 1qG and 16qL (Figure 6(b)). Currently, it is difficult to study this initial pattern of broad CNAs because these sorts of datasets are at a coarse level, where we can study these CNAs without also including 1qG and 16qL, which dominate the population of breast cancer. By identifying initial broad CNAs patterns in breast cancer, we may investigate if broad CNAs and somatic mutations present differently depending on initial pattern, further validate the mechanisms of early alterations proposed here, and study why different separation errors in normal breast cells lead to cancer (e.g., 1qG and 16qL) and why their compliment (e.g., chromosome 1 arm q broad loss and chromosome 16 arm q broad gain) do not. Furthermore, somatic copy number alteration patterns are not unique to breast cancer but appear in many cancer types [34, 42–56]. Future studies like this on other cancer types may reveal whether initial patterns of chromosome arms with autosomal broad CNAs are similar or unique between various cancers [56, 83, 84]. This may open the door to explore whether cancers emerge in similar or different ways and if they converge to similar or different patterns of broad CNAs as alterations accumulate.

The results of this work support that the number of autosomal broad CNAs must be considered in cancer research and cancer diagnosis. Moving forward, we plan to build on our understanding of how the dynamics of autosomal broad CNAs affect the progression of breast cancer and could contribute to more accurate diagnosis of individual patients. This work highlights how autosomal broad CNAs can be used as a framework to help expand our understanding of how early breast cancer evolved in vivo leading to a more symmetric response to an individual's current state of disease.

## Data Availability

The results shown here are in whole based upon data generated by the TCGA Research Network: https://www.cancer.gov/tcga. Specifically, the publicly available data from the TCGA-BRCA project may be found at https://portal.gdc.cancer.gov/projects/TCGA-BRCA.
